# A 3D *in Silico* Multi-Tissue Evolution Model Highlights the Relevance of Local Strain Accumulation in Bone Fracture Remodeling

**DOI:** 10.3389/fbioe.2022.835094

**Published:** 2022-03-31

**Authors:** Camille Perier-Metz, Laurent Corté, Rachele Allena, Sara Checa

**Affiliations:** ^1^ Julius Wolff Institute, Berlin Institute of Health at Charité—Universitätsmedizin Berlin, Berlin, Germany; ^2^ Centre des Matériaux, MINES Paris–PSL, Paris, France; ^3^ Chimie Moléculaire, Macromoléculaire et Matériaux, ESPCI Paris–PSL, Paris, France; ^4^ Laboratoire Mathématiques and Interactions J. A. Dieudonné, UMR 7351 CNRS, Université Côte d’Azur, Nice, France

**Keywords:** bone fracture remodeling, multi-tissue evolution, *in silico* modeling, *in vivo* validation, mechanobiology

## Abstract

Since 5–10% of all bone fractures result in non-healing situations, a thorough understanding of the various bone fracture healing phases is necessary to propose adequate therapeutic strategies. *In silico* models have greatly contributed to the understanding of the influence of mechanics on tissue formation and resorption during the soft and hard callus phases. However, the late-stage remodeling phase has not been investigated from a mechanobiological viewpoint so far. Here, we propose an *in silico* multi-tissue evolution model based on mechanical strain accumulation to investigate the mechanobiological regulation of bone remodeling during the late phase of healing. Computer model predictions are compared to histological data of two different pre-clinical studies of bone healing. The model predicted the bone marrow cavity re-opening and the resorption of the external callus. Our results suggest that the local strain accumulation can explain the fracture remodeling process and that this mechanobiological response is conserved among different mammal species. Our study paves the way for further understanding of non-healing situations that could help adapting therapeutic strategies to foster bone healing.

## 1 Introduction

Although bone usually has the capacity to heal spontaneously upon a given trauma, it is estimated that 5–10% of all fractures result in non-unions (Einhorn, 1995). A thorough understanding of the mechanisms driving the healing process can help to decipher non-healing situations and propose adequate therapeutic strategies. Bone fracture healing is a complex process that involves the coordination of multiple events. Traditionally, the bone healing process is described as following five overlapping stages: initial pro- and anti-inflammatory stages; soft callus formation; gradual mineralization towards a hard callus; and callus remodeling to restore the cortical bone geometry (Schell et al., 2017). The entire sequence of events has also been viewed from a different perspective, and the healing cascade has been proposed to consist of two distinct phases (Schindeler et al., 2008). First, during the anabolic stage, new bone and cartilage is formed, and during the catabolic phase, the cartilage is replaced by bone which is then further remodeled to restore the normal structure.

All these processes are known to be influenced by mechanical signals (Carter et al., 1988; Knecht et al., 2021), where it has been shown that specific strain and stress levels correlate with specific tissue type (bone, cartilage or fibrous tissue) formation or resorption. *In silico* fracture healing modelling has helped gaining knowledge on the mechanoregulation of the process, i.e., on the specific mechanical environment under which fracture healing takes place and how it drives tissue formation during the soft and hard callus phases (Prendergast et al., 1997; Claes et al., 1998; Lacroix and Prendergast, 2002; Isaksson et al., 2006). However, these bone healing computer models have generally failed at describing or ignored the remodeling stage of the healing, with bone being predicted in the whole fracture gap at the end of the healing period (Lacroix and Prendergast, 2002; Isaksson et al., 2008; Checa et al., 2011; Vetter et al., 2012; Borgiani et al., 2015; Wang and Yang, 2018). To our knowledge, only one model was used to simulate the bone fracture remodeling stage (Byrne et al., 2011), where the restoration of the bone cortices was predicted assuming the removal of the fracture fixation plate. However, remodeling has been observed to happen already in the presence of a fixation plate *in vivo* (Epari et al., 2006; Kruck et al., 2018; Wehrle et al., 2021).

Using a different approach, some *in silico* models have investigated bone remodeling as a response to controlled loading or un-loading of the bone (Beaupré et al., 1990; García et al., 2002; Hambli et al., 2011; Schulte et al., 2013). For instance, Doblaré et al., 2002; García et al., 2002 modelled the bone density evolution around hip implants or in a fracture, depending on various fracture fixation devices. They used a bone response model based on damage theory and an instantaneous mechanical stimulus to predict if bone would be formed, resorbed or keep the same density. Isaksson and others explained double cortex formation observed in mouse fracture healing by relating bone formation and resorption to thresholds in the local instantaneous strain energy density (Isaksson et al., 2009). In all studies, no other tissue taking part in the bone healing process (e.g., cartilage, fibrous tissue) was taken into account.

Here, we extended to 3D a previously developed 2D multi-tissue evolution model (Schmitt et al., 2016; Frame et al., 2017, 2018) and applied it to two different experimental set-ups (mouse and sheep long bone fracture healing) to investigate the mechanisms behind experimentally observed fracture remodeling patterns.

## 2 Materials and Methods

### 2.1 Computer Model of Tissue Remodeling

The computer model used to investigate tissue remodeling during bone fracture healing has been already described in 2D in the context of intact bone remodeling (Frame et al., 2017). Here, it was extended to 3D and implemented in COMSOL Multiphysics v.5.6 © (COMSOL AB, Sweden). The model consists of a mechanical finite element analysis, to determine the mechanical signals within the healing region, coupled with partial differential equations that simulate tissue evolution as a response to those signals. This model contains a continuous description of the tissue that corresponds to the averaging of all individual cell responses to their local mechanical environment.

The studied bone fracture geometries are discretized into tetrahedral finite elements (FE) for mechanical analysis and tissue evolution simulations: each FE of the callus (healing region) is hypothesized to contain tissue fractions of various types [fibrous tissue (F), cartilage (C), and bone (B)] and maturity [immature (I) and mature (M)], whose evolutions are subject to the rules described in the following sub-sections. The total tissue fraction $\varphi_{tot}$ is obtained by summing the different tissue fractions: $\varphi_{tot}=\varphi_{B,tot}+\varphi_{C,tot}+\varphi_{F,tot}$ , with $\varphi_{i,tot}=\varphi_{i}^{I}+\varphi_{i}^{M}$ for i=B, C, F (bone, cartilage or fibrous tissue).

#### 2.1.1 Biological Tissue Mechanosensing

The different biological tissue types are hypothesized to respond to the principal strains $\varepsilon_{k}$ (*k* = I, II or III). Based on previous studies (Carter et al., 1988; Claes and Heigele, 1999), cartilage is simulated to respond to the minimal principal strain (compressive) $\varepsilon_{III}$ only, fibrous tissue to the maximal one $\varepsilon_{I}$ (tensile) and bone to both $\varepsilon_{I}$ and $\varepsilon_{III}$ . The values of the local principal strains are normalized by the cortical bone yield strain ( $\varepsilon_{Y}$ ) and denoted by $\varepsilon_{k,N}.\;$ The responses are assumed quadratic as follows:

$$f_{i,k}\left(\varepsilon_{k}\right)=a_{i,k}\varepsilon_{k,N}^{2}+b_{i,k}|\varepsilon_{k,N}|+c_{i,k}$$ (1)

Where $a_{i,k},\;b_{i,k},\;c_{i,k}$ are tissue-specific coefficients defined in Table 1 (*i* = B, C or F). These quadratic functions become negative for low mechanical strains, representing a tissue resorption response. For a certain range of mechanical strains, the function increases with the strain magnitude, representing a tissue formation response. Tissue mechano-responses are assumed to be non-linear with respect to strain as it has been suggested to be more realistic than a linear response (Carter et al., 1988). Their parabolic shape additionally avoids the definition of a “lazy zone”, following recent literature which suggest that the lazy zone does not exist in the bone remodeling process (Christen et al., 2014; Razi et al., 2015; Weinkamer et al., 2019). The choice of the parameters for each tissue type defining the shape of the functions has been previously described (Frame et al., 2017). The tissue type i is assumed to respond to the strain accumulation following the function $t_{act,i}$ defined by:

$$\frac{\partial t_{act,i}}{\partial t}=\left(f_{i,I}\left(\varepsilon_{I}\right)+f_{i,III}\left(\varepsilon_{III}\right)\right)t_{act,i}^{bound}$$ (2)

With

$$t_{act,i}^{bound}=p\;\exp(-\frac{{\left(t_{act,i}-q\right)}^{2}}{2r²}))$$ (3)

a Gaussian distribution with *p*, *q*, *r* given in Table 1, ensuring a controlled rate of change for $t_{act,i}$ in over- or under-strained regions. Negative $t_{act,i}$ values lead to tissue resorption and positive ones to tissue formation and maturation. $t_{act,i}$ is further restricted to a tissue-specific activation range $\left[T_{i}^{min},T_{i}^{max}\right]$ to maintain realistic values for tissue formation or resorption rates:

$$T_{i}=\{\begin{array}{c} T_{i}^{\min}\;\;if\;t_{act,i}<T_{i}^{\min}\\ t_{act,i}\;\;if\;T_{i}^{\min}<t_{act,i}<T_{i}^{\max}\\ T_{i}^{\max\;}\;if\;t_{act,i}>T_{i}^{\max} \end{array}$$ (4)

**TABLE 1:** Computer model parameters [adapted from (Frame et al., 2017)].

Coefficients	Usage	Unit	Values
$\varepsilon_{Y}$	Bone yield strain	—	0.006
$a_{B,I}$ $b_{B,I}$ $c_{B,I}$	Bone mechano-response coefficients to normalised principal strain I	s^−1^	−6.25 6.25 −0.5625
$a_{B,III}$ $b_{B,III}$ $c_{B,III}$	Bone mechano-response coefficients to normalised principal strain III	s^−1^	−6.25 7.5 −0.75
$a_{C,III}$ $b_{C,III}$ $c_{C,III}$	Cartilage mechano-response coefficients to normalised principal strain III	s^−1^	−12.5 25 −11.5
$a_{F,I}$ $b_{F,I}$ $c_{F,I}$	Fibrous tissue mechano-response coefficients to normalised principal strain I	s^−1^	−5 12.5 −6.8125
p q r	$t_{act}^{bound}$ coefficients for all tissue types	s	110,000 20,000
$T_{B}^{\min}\;T_{B}^{\max}$	Activation time range for bone	s	−20000 50,000
$T_{C,F}^{min}\;T_{C,F}^{max}$	Activation time range for cartilage and fibrous tissue	s	−10000 50,000
$k^{M}\;l^{M}\;m^{M}$	$T^{M}$ (maturation) coefficients for all tissue types	s	130,000 10,000
$k_{B}^{R}\;l_{B}^{R}\;m_{B}^{R}$	$T_{B}^{R}$ (resorption) coefficients for bone	s	1 −15,000 10,000
$k_{C}^{R}\;l_{C}^{R}\;m_{C}^{R}$	$T_{C}^{R}$ (resorption) coefficients for cartilage	s	1 −5,000 10,000
$k_{F}^{R}\;l_{F}^{R}\;m_{F}^{R}$	$T_{F}^{R}$ (resorption) coefficients for fibrous tissue	s	1 −5,000 10,000
$T_{B,GT}$ $T_{C,GT}\;T_{F,GT}$	Normalisation constants for tissue formation	s	20,000 20,000 10,000
λ Φ	Diffusion tensor coefficients	m^3^.s^−1^	1e-10 1e-8
$\alpha_{B}\;\alpha_{C}\;\alpha_{F}$	Tissue formation rate coefficients	m^3^.s^−1^	4e-5 1e-5 1e-5
$\beta_{B}\;\beta_{C}\;\beta_{F}$	Resorption rate coefficients	m^3^.s^−1^	1e-7 1e-7 1e-7
$\gamma_{B}\;\gamma_{C}\;\gamma_{F}$	Maturation rate coefficients	m^3^.s^−1^	2e-6 1e-5 1e-3
$E_{B}^{I}\;E_{C}^{I}\;E_{F}^{I}$	Immature tissue Young’s moduli	MPa	10 1 1
$E_{B}^{M}\;E_{C}^{M}\;E_{F}^{M}$	Mature tissue Young’s moduli (sheep)	MPa	20,000,100 1
$E_{B}^{M}\;E_{C}^{M}\;E_{F}^{M}$	Mature tissue Young’s moduli (mouse)	MPa	5000^a^ 100 1

a Borgiani et al. (2015).

The tissue-specific responses $T_{i}$ (*i* = B, C, F) serve to define tissue formation (Eq. 5), resorption (Eq. 6) and maturation (Eq. 7) functions (superscripts G, R, M, respectively):

$$T_{i}^{G}=\{\begin{array}{c} \frac{T_{i}}{T_{i,GT}}\;if\;T_{i}>0\;\\ 0\;otherwise \end{array}$$ (5)

$$T_{i}^{R}=k_{i}^{R}\exp)-\frac{(T_{i}-l_{i}^{R}){\;}^{2}}{(2m_{i}^{R})²})$$ (6)

$$T_{i}^{M}=k^{M}\exp(-\frac{(T_{i}-l^{M}){\;}^{2}²}{(2m^{M})²})$$ (7)

Normalization constant for tissue formation $T_{i,GT}$ and coefficients (*k*, *l*, *m*) defining the Gaussian distributions for resorption and maturation are defined in Table 1. These different functions model the fact that tissue formation, resorption and maturation happen under different ranges of accumulated strain. In particular, tissue formation is zero (no formation happening) for negative values of $T_{i}$ and scales linearly with positive $T_{i}$ , to describe how immature tissue forms faster with increasing $T_{i}$ and thus replicate the time lag during bone primary mineralization.

#### 2.1.2 Tissue Evolution Equations

**FIGURE 1:**
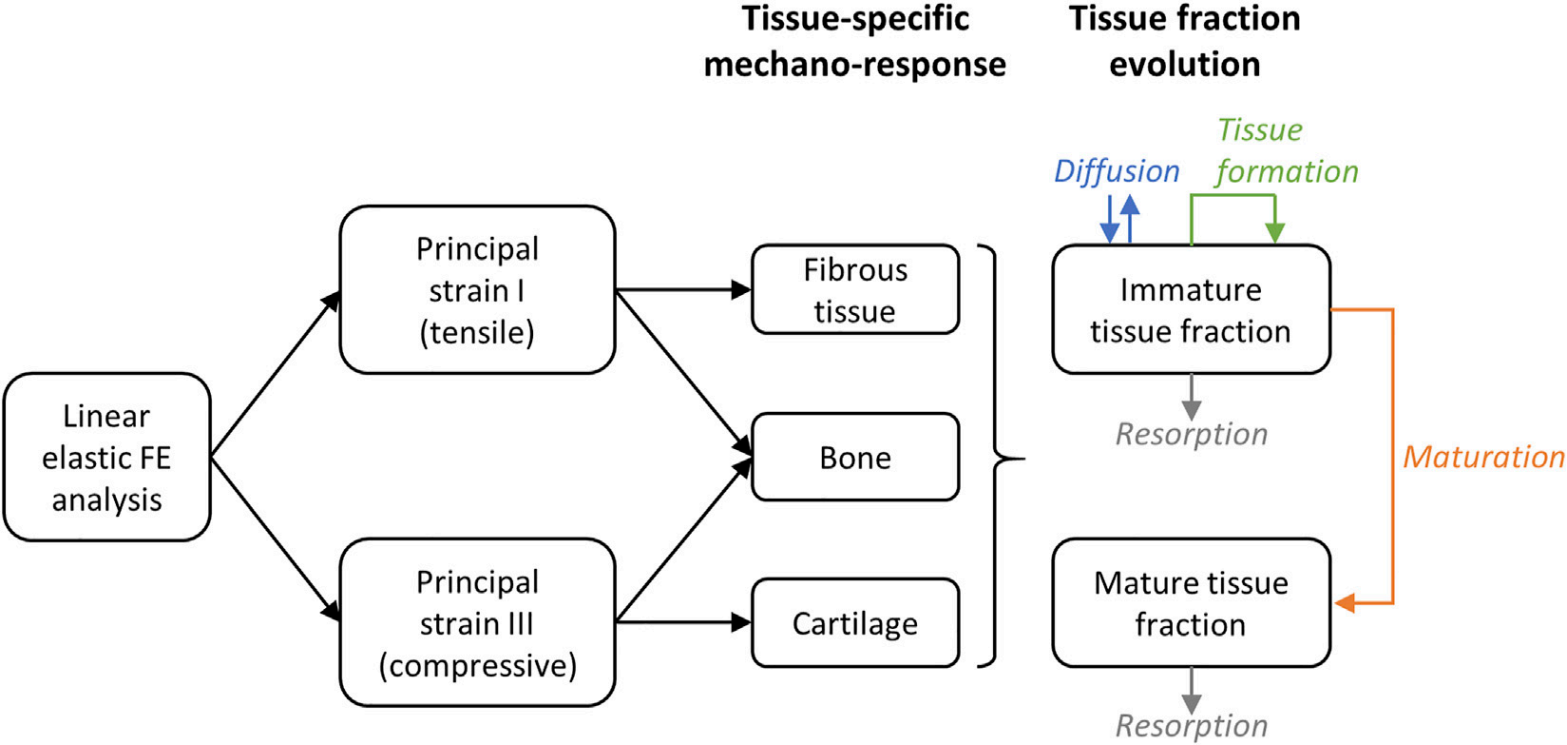
Principle of the mechano-sensing and tissue fraction evolution simulation. On the right (tissue fraction evolution), the direction of the arrow indicates if the contribution is positive (arrow pointing towards the box) or negative (arrow pointing away from the box).

The different immature and mature tissue fractions evolve depending on the following mechanisms: diffusion, positive contribution of tissue formation and negative contribution of resorption and maturation for the immature tissue fractions (Eq. 8); positive contribution of maturation and negative contribution of resorption for the mature tissue fractions (Eq. 9) (Figure 1). In particular, time was taken into account in the FE simulations to determine changes in the mechanical strains within the healing region due to the formation, resorption and maturation of the different tissues over time. In each time step, the material properties of the FE model were updated based on the predicted tissue fractions and a mechanical analysis was performed to determine the mechanical principal strains. The evolution is implemented by means of diffusion-reaction equations for tissue type i (*i* = B, C or F):

$$\frac{\partial \varphi_{i}^{I}}{\partial t}=\left[\left(1-\varphi_{tot}\right)D\Delta \varphi_{i}^{I}\right]+\;\left[\alpha_{i}\left(1-\varphi_{tot}\right)\varphi_{tot}T_{i}^{G}\right]-\left[\beta_{i}\varphi_{i}^{I}T_{i}^{R}\right]-\left[\gamma_{i}\varphi_{i}^{I}T_{i}^{M}\right]$$ (8)

$$\frac{\partial \varphi_{i}^{M}}{\partial t}=-\left[\beta_{i}\varphi_{i}^{M}T_{i}^{R}\right]+\left[\gamma_{i}\varphi_{i}^{I}T_{i}^{M}\right]$$ (9)

The immature tissue diffusion term $\left(1-\varphi_{tot}\right)D\Delta \varphi_{i}^{I}$ is defined as the product of the 3D diffusion tensor **D** and the Laplacian of the immature tissue fraction, corrected by the factor $1-\varphi_{tot}$ to account for available space, where D reads: $D=\;\lambda I+\Phi \left[\sqrt{\left|\varepsilon_{I}\right|}\theta_{I}⊗\theta_{I}+\sqrt{\left|\varepsilon_{II}\right|}\theta_{II}⊗\theta_{II}+\sqrt{\left|\varepsilon_{III}\right|}\theta_{III}⊗\theta_{III}\right]$ with ⊗ the tensor product; $\theta_{k}$ the direction of principal stress k; **I** the identity tensor; λ and Φ diffusion coefficients defined in Table 1.

The immature tissue formation term $\alpha_{i}\left(1-\varphi_{tot}\right)\varphi_{tot}T_{i}^{G}$ is proportional to the tissue formation function $T_{i}^{G}$ , $\varphi_{tot}$ as all tissue types are assumed to produce new immature tissue, and the available space $\left(1-\varphi_{tot}\right)$ , with a proportionality factor $\alpha_{i}$ (tissue-specific formation rate, Table 1). The tissue resorption terms $\beta_{i}\varphi_{i}^{I}T_{i}^{R}$ and $\beta_{i}\varphi_{i}^{M}T_{i}^{R}$ are proportional to the resorption function $T_{i}^{R}$ and the immature (resp. mature) tissue fraction with a factor $\beta_{i}$ (tissue-specific resorption rate, Table 1). The tissue maturation term $\gamma_{i}\varphi_{i}^{I}T_{i}^{M}$ is proportional to the maturation function $T_{i}^{M}$ and the immature tissue fraction with a factor $\gamma_{i}$ (tissue-specific maturation rate, Table 1).

#### 2.1.3 Mechanical Analysis

The diffusion-reaction equations for the different tissue type evolutions are coupled to a mechanical analysis providing the principal strain values and stress directions. All materials are assumed to be linear isotropic elastic materials following the Hooke’s law. Each tissue type in immature and mature states is attributed specific Young’s moduli (Table 1) and a Poisson’s ratio of 0.3. A rule of mixtures is used to compute the FE Young’s modulus by averaging the material properties of its various tissue fractions: $E=\underset{i}{\sum }\left(\varphi_{i}^{I}E_{i}^{I}+\varphi_{i}^{M}E_{i}^{M}\right)$ where $E_{i}^{I}$ (resp. $E_{i}^{M}$ ) is the Young’s modulus of the immature (resp. mature) tissue i (*i* = B, C or F). Other specific material properties and loading and boundary conditions of the FE models are given in Sections 2.2.3, 2.2.4.

### 2.2 Application to Fracture Healing Experiments

#### 2.2.1 Experimental Set-Ups

The multi-tissue evolution model was applied to a mouse (Kruck et al., 2018) and a sheep fracture healing experiments (Epari et al., 2006).

**FIGURE 2:**
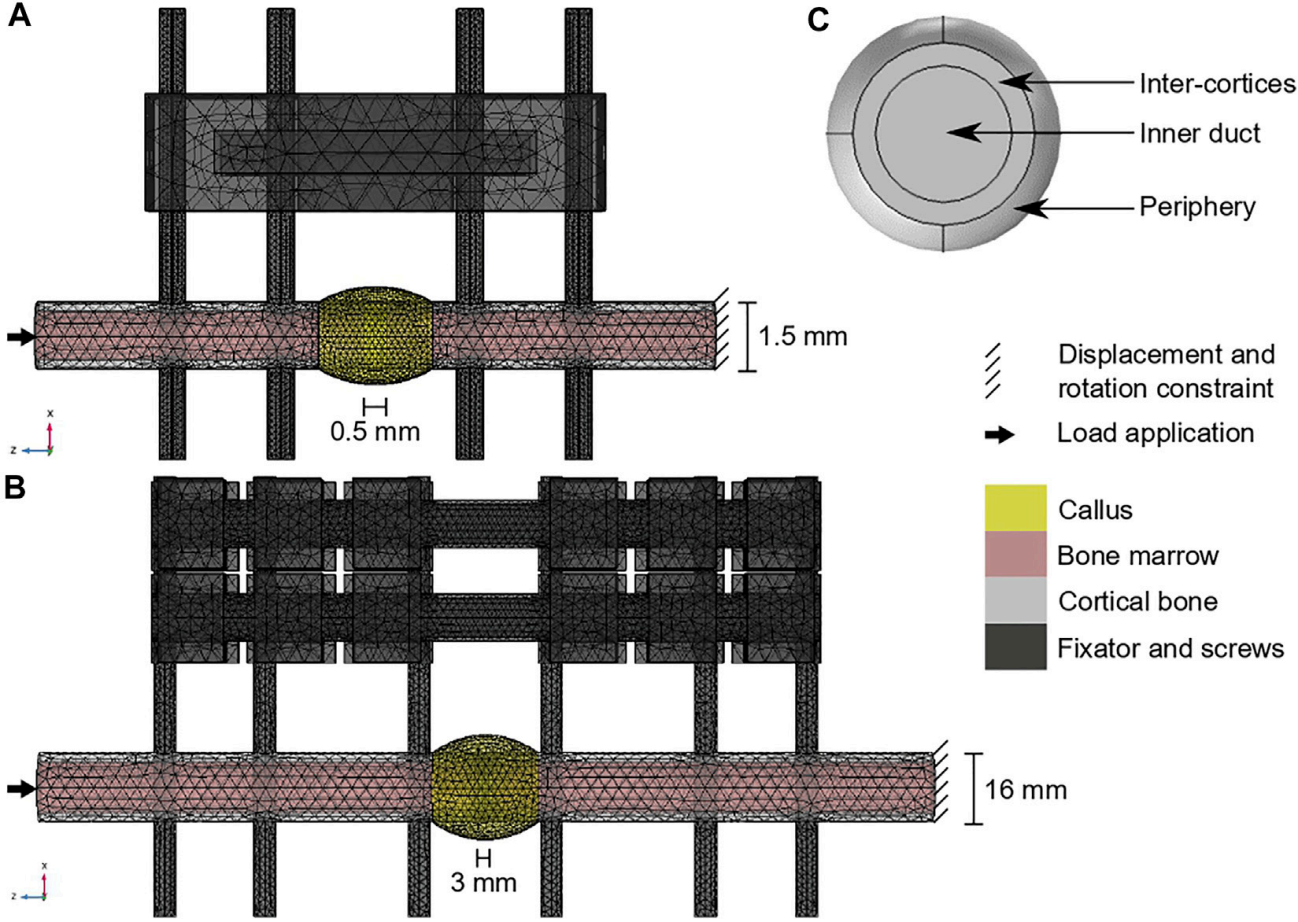
Computer model set-ups: **(A)** Mouse fracture healing geometry; **(B)** sheep fracture healing geometry; **(C)** region of interest definition in the callus radial view (shown in yellow on a and b). The color code for **(A,B)** is given on the right.

Briefly, in the mouse experiment, a 0.5-mm osteotomy was performed at the femoral mid-shaft in adult mice. The defect was fixed using a polyether ether kethone (PEEK) external fixator and four titanium screws (Figure 2A). Follow-up histology (mid-sagittal cut with Movat-Pentachrome staining) was performed 7, 14 and 21 days post-surgery to reveal bone in yellow, cartilage in green and fibrous tissue in red (Kruck et al., 2018).

In the sheep experiment, a 3-mm osteotomy was performed at the tibial mid-shaft in adult sheep. The defect was fixed using a stainless-steel external fixator and six steel screws (Figure 2B). Follow-up histology (mid-sagittal cut with Safranin Orange/van Kossa staining) was performed 2, 3, 6 and 9 weeks post-surgery to reveal bone in black, cartilage in dark red and fibrous tissue in bright red (Epari et al., 2006).

#### 2.2.2 Computer Model Geometries

Both experimental set-ups were reproduced in COMSOL v.5.6 to simulate tissue evolution in the callus (regenerating region). Intact bone, marrow cavity, fixator and screws were included in the models. Fixator shapes and dimensions were taken from the experiments. Simulation times were chosen to reproduce the experimentally observed tissue remodeling and were then related to physiological times based on the analysis results.

For the mouse model (Figure 2A), the intact bone extremities were modelled as cylinders of radius 0.75 mm containing the marrow cavity of radius 0.55 mm (Borgiani et al., 2015). The screws had a radius of 0.3 mm and a length of 10 mm. The callus was obtained by rotating a circle arc of height 0.3 mm around the 0.5-mm defect and covering 1 mm of the intact bone extremities. All parts were meshed with tetrahedral elements of following average sizes: 0.3 mm for the intact cortical bone extremities and the bone marrow cavities, 0.2 mm for the screws, 0.6 mm for the fixator, 0.16 mm for the callus inner duct, inter-cortices and periphery regions. The simulation was run for 3000 h (simulation time).

For the sheep model (Figure 2B), the intact bone extremities were modelled as cylinders of radius 8 mm containing the marrow cavity of radius 6 mm (Checa et al., 2011). The screws had a radius of 2.5 mm and a length of 100 mm. The callus was obtained by rotating a circle arc of height 4 mm around the 3-mm defect and covering 1 cm of the intact bone extremities. All parts were meshed with tetrahedral elements of following average sizes: 3.3 mm for the intact cortical bone extremities, 3.5 mm for the bone marrow cavities, 2.5 mm for the screws, 3.8 mm for the fixator, 1.1 mm for the callus inner duct and inter-cortices regions and 2.1 mm for the callus periphery. The simulation was run for 6000 h (simulated time).

#### 2.2.3 Material Properties

In addition to the regenerating tissue material properties (Table 1), intact cortical bone, bone marrow, steel (sheep screw and fixator), PEEK (mouse fixator) and titanium (mouse screws) were assumed to be isotropic linear elastic materials, with properties defined in Table 2.

**TABLE 2:** Material properties of the fixators, screws and non-regenerating tissues.

Material	Young’s modulus (MPa)	Poisson’s ratio
Mouse cortical bone	5,000^a^	0.3^b^
Sheep cortical bone	17,000^b^	0.3^b^
Bone marrow	2^b^	0.167^b^
PEEK	3,800^a^	0.3^a^
Titanium	170,000^a^	0.3^a^
Steel	210,000^b^	0.3^b^

a Borgiani et al.(2015).

b Checa et al.(2011).

#### 2.2.4 Loading and Boundary Conditions

In both set-ups, the distal end of the bone was clamped (all translation and rotation degrees of freedom were set equal to 0). For the mouse (average weight 0.025 kg), 1.5 N compression, 0.3 N lateral-medial bending and 0.3 N antero-posterior bending were applied on the proximal end of the bone (Borgiani et al., 2015). For the sheep (average weight 60 kg), 1200 N compression and 75 N antero-posterior bending were applied on the proximal end of the bone (Duda et al., 1997; Checa et al., 2011).

#### 2.2.5 Initial Tissue Distributions

The multi-tissue evolution model aimed at investigating the experimentally observed late remodeling phase in the fracture healing experiments. Therefore, the 14-day histology data (mouse, 7 samples) and 6-week histology data (sheep, 1 sample) were segmented and quantified for mature tissue content. The average mature tissue fractions were computed to define the initial tissue distribution in the callus at the start of the remodeling simulation, in the three regions of interest (ROIs) defined in Figure 2C: inner duct, inter-cortices, periphery (Table 3). The immature tissue fractions were assumed to be equal to 5% initially as they could not be visualized in the experimental data.

**TABLE 3:** Initial tissue fractions in the regions of interest, computed from the quantification of the 14-day and 6-week histology data for mouse and sheep, respectively; the immature tissue fractions were all assumed to be equal to 5% initially.

Tissue	Mouse			Sheep		
	Inner duct	Inter-cortices	Periphery	Inner duct	Inter-cortices	Periphery
Immature bone	0.05	0.05	0.05	0.05	0.05	0.05
Mature bone	0.39	0.45	0.16	0.15	0.65	0.45
Immature cartilage	0.05	0.05	0.05	0.05	0.05	0.05
Mature cartilage	0.32	0.34	0.16	0.1	0.06	0.1
Immature fibrous tissue	0.05	0.05	0.05	0.05	0.05	0.05
Mature fibrous tissue	0.06	0.05	0.11	0.32	0.05	0.2

### 2.3 Computer Model Output Analysis

Histology-like images were derived from the computer model predictions at start and end of the simulation corresponding to 14 and 21 days (mouse) and 6 and 9 weeks (sheep) post-surgery: cartilage density was plotted in green (mouse) or red (sheep) shades, fibrous tissue density in red shades, bone density in yellow (mouse) or black (sheep) shades. In addition, to investigate the observed tissue evolution, the distribution of the minimal and maximal principal strains and the bone activation functions T_B_ were plotted over time in the mid-sagittal plane. Besides, the average mature bone, cartilage and fibrous tissue fractions were quantified over time in the three ROIs (Figure 2C).

## 3 Results

### 3.1 Mouse Fracture Healing

**FIGURE 3:**
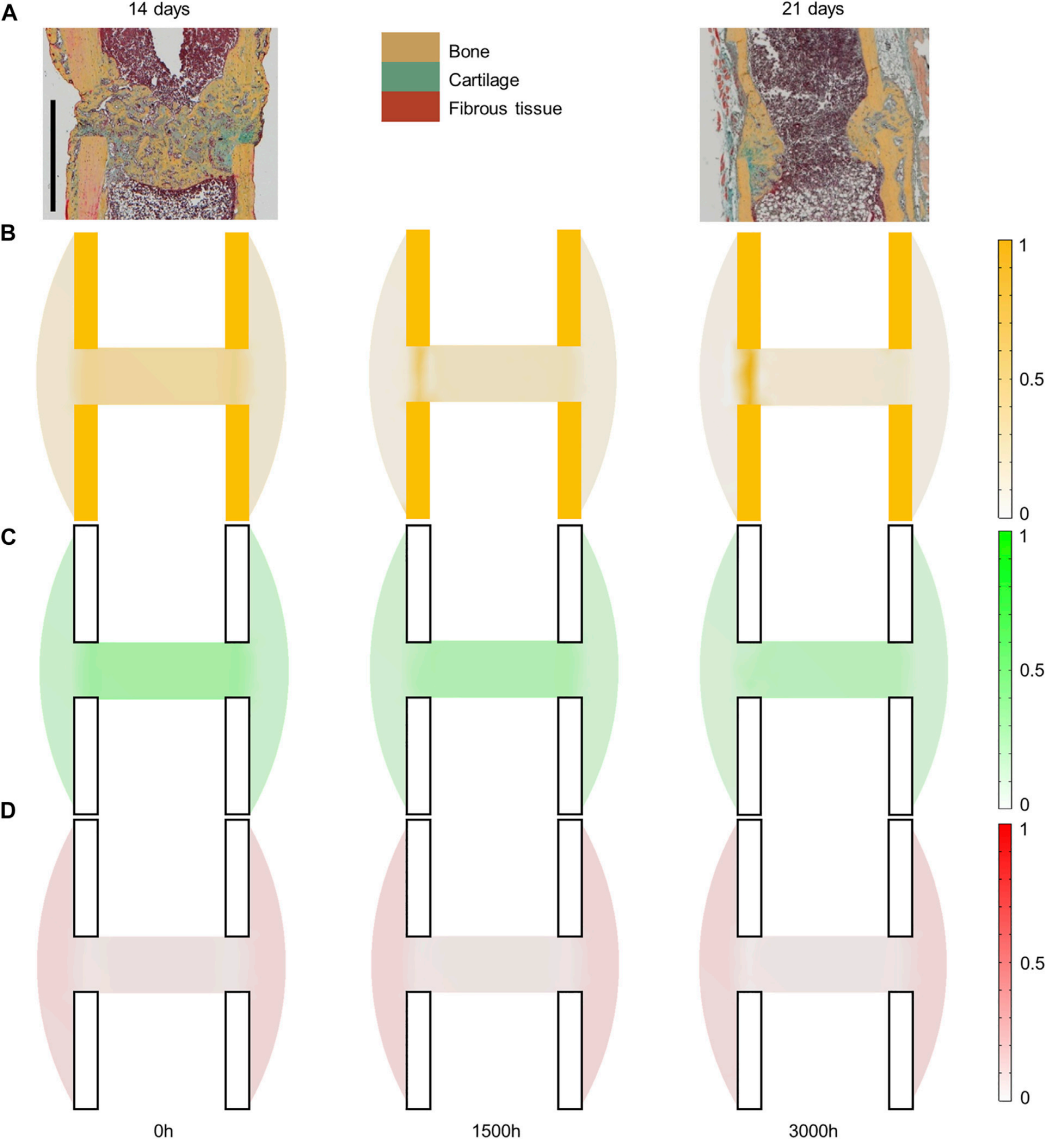
Mouse fracture tissue distribution in the mid-sagittal plane: **(A)** experimental 14- and 21-day histology (image courtesy: Bettina Kruck); **(B)** predicted mature bone fraction at 0, 1,500 and 3000 h; **(C)** predicted mature cartilage fraction at 0, 1,500 and 3000 h; **(D)** predicted mature fibrous tissue fraction at 0, 1,500 and 3000 h. Scale bar: 1 mm; the fixator was placed on the right of all images (medial side).

**FIGURE 4:**
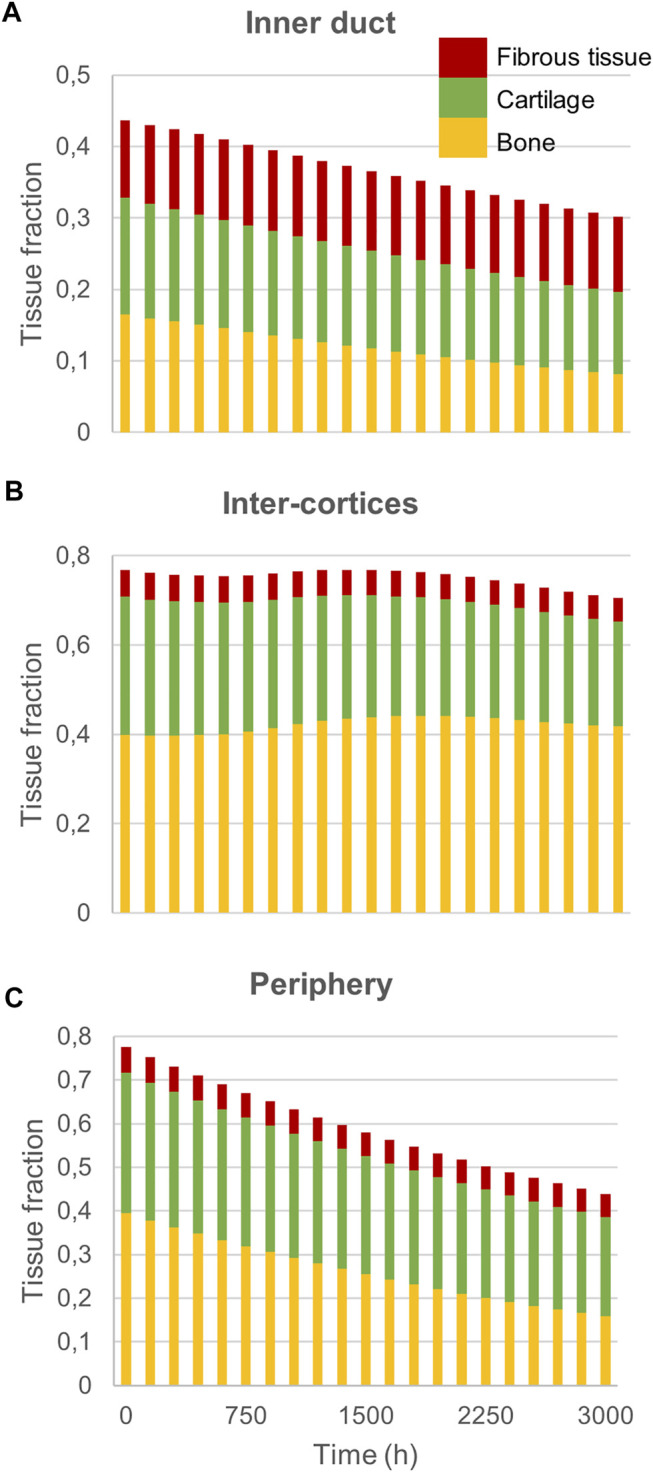
Mouse fracture quantification: Mature bone, cartilage and fibrous tissue fraction evolution over time in **(A)** the inner duct; **(B)** the inter-cortices and **(C)** the periphery.

Experimentally, mice experienced tissue remodeling between 14 and 21 days post-surgery (Figure 3A), with a re-opening of the bone marrow cavity. The simulation started from a homogeneous high-density bone in the inner duct and inter-cortices (similar to the histology 14 days post-surgery) and led to denser bone formation in the inter-cortices and resorption in the inner duct and the periphery (Figure 3B). The addition of one intermediate time-point (Figure 3B) and the quantification over time (Figure 4) gave more insight into the healing process: the bone volume fraction decreased over time in the inner duct and in the periphery. In the inter-cortices, the bone volume fraction stagnated, slightly increased, in particular in the side opposite to the fixation plate, and slightly decreased again (Figures 3B, 4). Cartilage and fibrous tissue volume fractions decreased only slightly over time (Figures 3C,D, 4).

**FIGURE 5:**
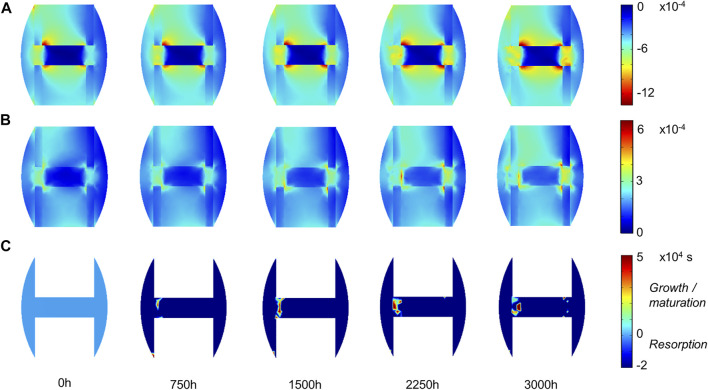
Mouse fracture tissue mechano-sensation in mid-sagittal plane: **(A)** minimal principal strain $\varepsilon_{III}$ ; **(B)** maximal principal strain $\varepsilon_{I}$ ; **(C)** bone-specific strain response function (restricted to its activation range). Other tissue-specific strain response functions are not plotted, as they were constantly and uniformly equal to their minimal value (thus leading to resorption).

The principal strain values remained lower than 0.12% in compression and 0.06% in tension (Figures 5A,B), leading to fibrous tissue and cartilage mechano-responses that were always minimal, thus yielding resorption of those tissue types (Figures 3C,D). The bone mechano-response was positive (bone formation) mainly in the inter-cortices region, and rather on the side opposite to the external fixator (left on the figures), at late time-points (Figure 5C). The time evolution of the bone mechano-response corresponded thus to the observed stagnation and slight increase in bone volume fraction in the inter-cortices (Figures 3B, 4). In the inner duct and the periphery, the response was always negative, leading to bone resorption (Figures 4, 5). Bone resorption was associated with absolute minimal and maximal principal strain levels of <0.08% and <0.03%, respectively, whereas bone formation was associated with absolute minimal and maximal principal strain levels of approximately 0.08 and 0.03%, respectively.

### 3.2 Sheep Fracture Healing

**FIGURE 6:**
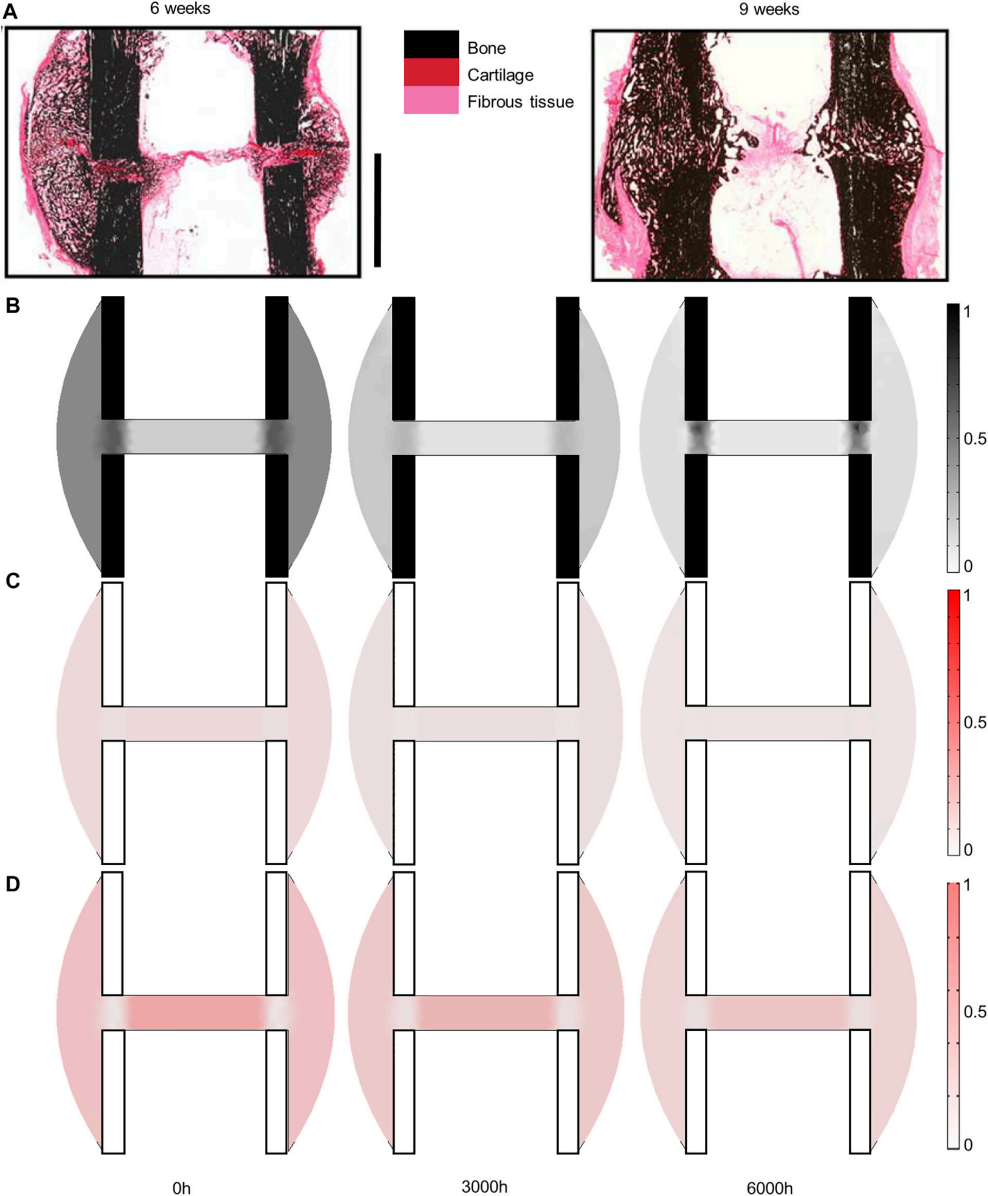
Sheep fracture tissue distribution in the mid-sagittal plane: **(A)** experimental 6- and 9-week histology (reproduced with permission from (Epari et al., 2006) Copyright © 2005 Elsevier Inc.); **(B)** predicted mature bone fraction at 0, 3,000 and 6000h; **(C)** predicted mature cartilage fraction at 0, 3,000 and 6000h; **(D)** predicted mature fibrous tissue fraction at 0, 3,000 and 6000 h. Scale bar: 10 mm; fixator was placed on the right of all images (medial side).

**FIGURE 7:**
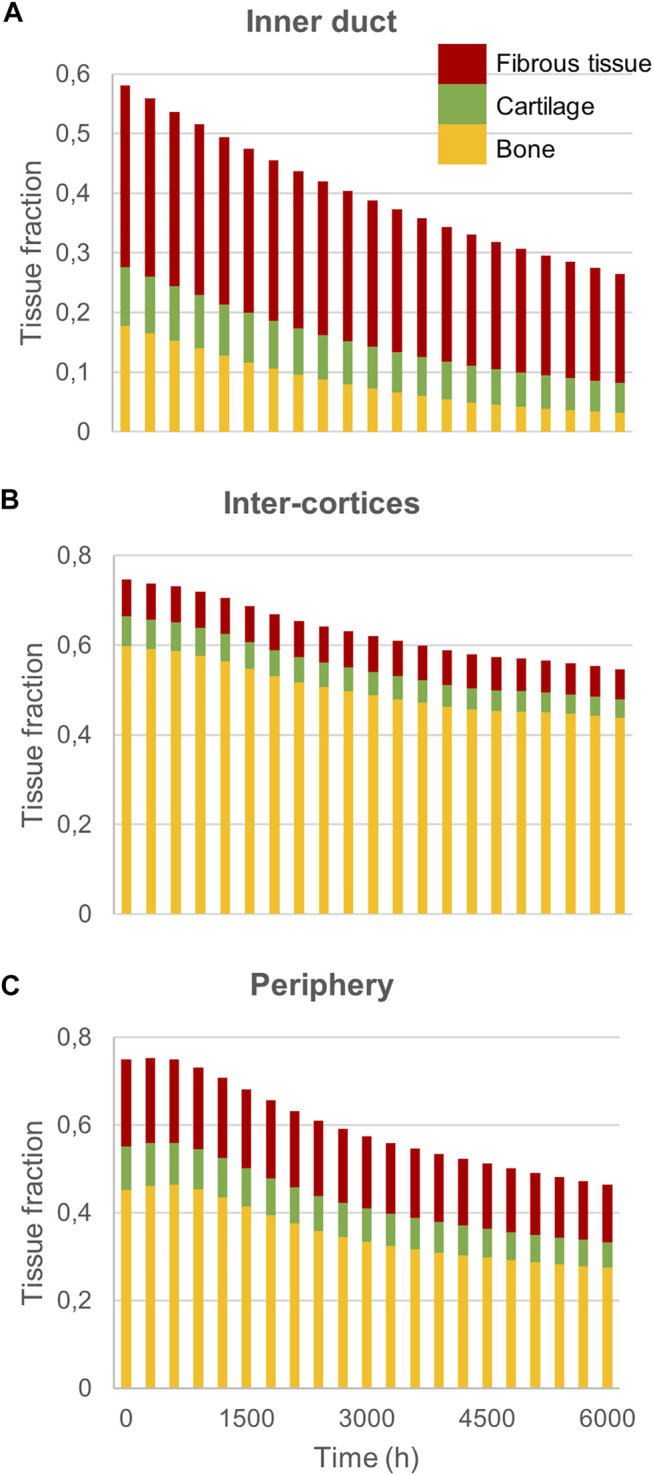
Sheep fracture quantification: Mature bone, cartilage and fibrous tissue fraction evolution over time in **(A)** the inner duct; **(B)** the inter-cortices and **(C)** the periphery.

Experimentally, sheep experienced remodeling of the hard callus between 6 and 9 weeks post-surgery (Figure 6A), with a re-opening of the bone marrow cavity and resorption of the peripheral callus. The simulation started from a homogeneous high-density bone in the inter-cortices and intermediate-density in the periphery (similar to the histology 6 weeks post-surgery) and led to slightly less dense bone formation in the inter-cortices and stronger resorption in the periphery and the inner duct regions (Figure 6B). The addition of one intermediate time-point (Figure 6B) and the quantification over time (Figure 7) gave more insight into the healing process: the bone volume fraction decreased over time in the inner duct, while it increased slightly and decreased again in the periphery. In the inter-cortices, the initially very high bone volume fraction first decreased and then reached a plateau at ca. 45%. Cartilage and fibrous tissue volume fractions decreased slowly over time (Figures 6C,D, 7).

**FIGURE 8:**
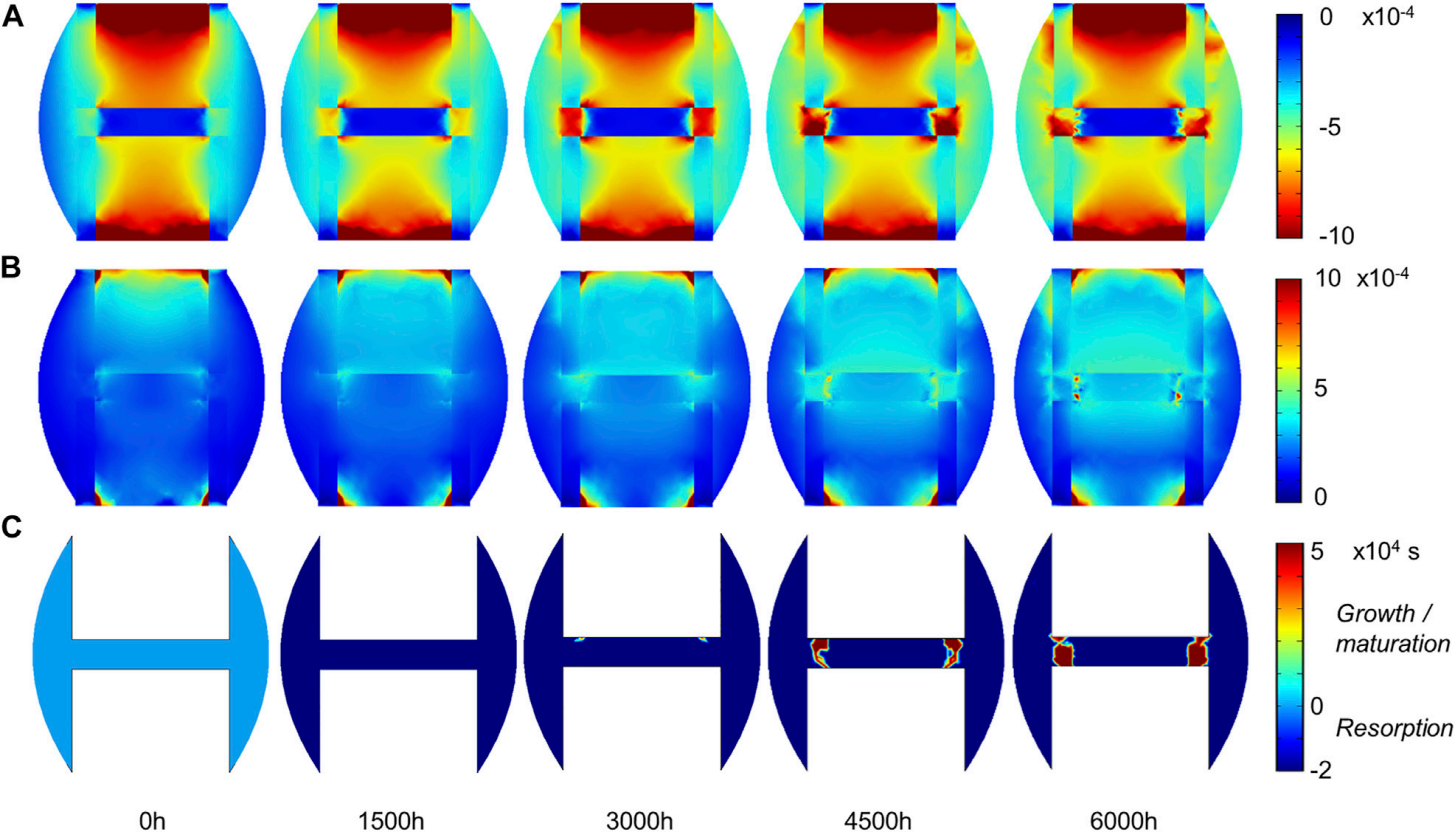
Sheep fracture tissue mechano-sensation in mid-sagittal plane: **(A)** minimal principal strain. $\varepsilon_{III}$ ; **(B)** maximal principal strain $\varepsilon_{I}$ ; **(C)** bone-specific strain response function (restricted to its activation range). Other tissue-specific strain response functions were not plotted, as they were constantly and uniformly equal to their minimal value (thus leading to resorption).

The principal strain values remained lower than 0.1% in compression and tension (Figures 8A,B), leading to fibrous tissue and cartilage mechano-responses that were always minimal, thus yielding resorption of those tissue types (Figures 6C,D). The bone mechano-response was positive only in the inter-cortices region and at late time-points; it was negative during the rest of the simulation as the regenerating zone was very stiff, with low strain levels (Figures 8A,B). The time evolution of the bone mechano-response corresponded thus to the observed decrease and stagnation in bone volume fraction in the inter-cortices, and the decrease in the inner duct and periphery (Figures 6B, 7). Bone resorption was associated with absolute minimal and maximal principal strain levels of <0.05% and <0.01%, respectively, whereas bone formation was associated with absolute minimal and maximal principal strain levels of approximately 0.05–0.1 and 0.03%, respectively.

## 4 Discussion

We presented here a multi-tissue evolution computer model that proved capable of predicting experimentally observed fracture healing remodeling in mice (Kruck et al., 2018) and sheep (Epari et al., 2006) assuming a tissue response to strain accumulation over time. Aim of the simulation studies was to understand the principles behind the observed tissue remodeling in the 21-day (respectively 9-week) data for the mouse (resp. sheep), starting from the 14-day (resp. 6-week) data. In particular, the computer model predicted callus resorption and re-opening of the bone marrow cavity as observed experimentally.

Previous *in silico* fracture healing studies were able to describe the soft callus and hard callus phases by coupling tissue formation to the local mechanics in the healing region (Lacroix and Prendergast, 2002; Isaksson et al., 2008; Checa et al., 2011; Vetter et al., 2012; Borgiani et al., 2015; Wilson et al., 2017; Wang and Yang, 2018). However, they did not predict the last remodeling stage and bone was predicted to remain in the complete bone marrow cavity at the end of the simulation. A further *in silico* study validated a bone remodeling model against mouse fracture healing experiments (Isaksson et al., 2009), which could successfully predict the double cortex formation observed in late-stage mouse healing. However, this study did not include other tissue types in the process and focused only on the mouse where this specific phenomenon is observed. An *in silico* fracture healing model was used to simulate the callus remodeling phase in a human tibia 3-mm defect (Byrne et al., 2011). Although the predictions were not compared to experimental data, this model predicted the restoration of the cortices and the removal of the cartilage phase by using a mechanoregulation theory based on octahedral shear strain and relative fluid velocity. Interestingly, the fracture remodeling was predicted only once the fixator was removed from the simulation (Byrne et al., 2011). In our study, the experimental data suggested that remodeling happened in the presence of the fixator, what could be simulated by our model assuming a response to mechanical strain accumulation over time.

Other bone healing computer models have investigated bone remodeling, but they did not include fibrous tissue or cartilage fractions (Doblaré et al., 2002; García et al., 2002; Hambli et al., 2011; Goda et al., 2016; Mercuri et al., 2016). Multi-tissue evolution constitutes therefore a unique feature of the model used in this study (Frame et al., 2017), making it particularly suitable for fracture healing description where multiple tissues are involved (Schindeler et al., 2008). In particular, the investigation of the mechanical stimuli over time revealed negative fibrous tissue and cartilage response functions, leading to their resorption; and specific patterns in the bone response function leading to the observed higher bone density in the region joining the intact cortices. In the sheep, strain levels leading to bone resorption were lower than 100 microstrains (µε) in tension and 500 µε in compression, whereas bone formation happened under 500–1000 µε compression but very low tension (<300 µε). In the mouse, thresholds between bone resorption and formation were around 300 µε in tension and 800 µε in compression. Similar value ranges have been used for the strain energy density threshold in bone remodeling literature: 1000 µε in (Dunlop et al., 2009), 500 µε in (Isaksson et al., 2009), 700–1000 µε in (Schulte et al., 2013); Razi and others also found a lower threshold for bone response in tension (300 µε) compared to compression (1200 µε) (Razi et al., 2015). Here, these values are no exact thresholds as the accumulation of strain over a few hours was taken into account as a mechano-response. Indeed, when strain levels increased after the initial bone resorption in the inter-cortices region in the sheep (3000 h) and the mouse (1500 h), the bone mechano-response remained negative and would turn positive only later (Figures 5C, 8C). The implementation of this hysteresis rule avoided the oscillations sometimes seen in bone healing predictions that rely only on one threshold between bone resorption and formation (Perier-Metz et al., 2020). More importantly, this is a way to take into account the accumulation of damage and micro-cracks taking part in the bone remodeling process (Carter et al., 1988; Mori and Burr, 1993; Prendergast and Taylor, 1994; Doblaré et al., 2002; Martin and Seeman, 2008). Previous computer models of bone remodeling based on strain accumulation have been able to predict the femur head bone density distribution (Frame et al., 2018) and healing patterns in a mandible (Schmitt et al., 2016); however, other mechanical signals have also proven to be valid, e.g., the strain energy density (Schulte et al., 2013). In general, a consensus about the mechanical stimulus driving the bone remodeling process does not exist (Webster and Müller, 2011; Weinkamer et al., 2019).

We applied the same computer model in two different species (mouse and sheep) and found no inter-species difference. This finding contrasts with previous results in fracture healing simulations where different mechanosensitivity levels were hypothesized to explain fracture healing patterns in the mouse, rat and sheep (Checa et al., 2011; Borgiani et al., 2015). Here, adapted material properties, geometries, loading conditions and initial tissue distribution (intermediate fracture healing stage) were sufficient to reproduce each animal’s specific tissue patterns. Experimental studies also suggested similar remodeling behaviors among various mammal species and humans (Garetto et al., 1995; Christen et al., 2014).

The computer model used in this study had several limitations and simplifications. First, the simulation time did not match the biological time, although tissue formation rates were derived from experimental literature. Remodeling was observed in 7 days in the mouse and 3 weeks in the sheep, whereas *in silico* approximately 88 “model days” were needed for the mouse and 225 for the sheep; the computer model prediction time should therefore be divided by approximately 10–12 to describe the real biological time. However, the ratio between both animals was similar *in silico* and *in vivo*, with a remodeling process happening ca. 3 times faster in the mouse than in the sheep. Therefore, we discarded the simulation time as having any biological meaning and used it only for analysis purposes of the tissue evolution in the healing process. Fibrous tissue and cartilage remnants were predicted, for instance in the inner gap region where the bone marrow cavity should actually form back. These wrong predictions are likely due to the absence of modelling the restoration of the bone marrow in the fracture gap and of other surrounding tissues (for the periphery region). A more complete model could include these tissues. A further limitation consisted in restricting the regenerating region to the callus; in fact, experimental observations suggest that the intact cortical bone is also remodeled during fracture healing (Vetter et al., 2010; Wehrle et al., 2021); it could thus be included in the diffusion-formation-resorption process in future studies. Here, mechano-response was hypothesized to be driven solely by principal strains, as hypothesized in previous bone remodeling theories (Frost, 1996); however, combinations of shear strains and relative fluid velocity (Prendergast et al., 1997) or minimal principal strain and hydrostatic stress (Claes and Heigele, 1999) have been suggested as mechanical stimuli in fracture healing. A more comprehensive mechanoregulation theory might better describe fibrocartilage resorption over the course of healing. Moreover, isotropic linear elastic descriptions were chosen for the different materials; poroelasticity and anisotropy of the bone (Giorgio et al., 2016), and hyperelasticity of the cartilage and fibrous tissue (Freutel et al., 2014) could be included in future studies for more realistic tissue descriptions. Lastly, the tissue mechano-response and the evolution equations require several parameters that are derived from literature or experimental values; a parameter sensitivity analysis was performed to identify the most critical parameters for the predictions (Supplementary Material S1). A few bone mechano-response parameters (b_B,III_ and c_B,III_, b_B,I_ and c_B,I_, m_B_^M^, m_B_^R^) were found to influence strongly the predicted bone density (Supplementary Figure S2). The values for b_B,III_, c_B,III_, b_B,I_ and c_B,I_, relating the bone mechano-response to the third and first principal strain values, respectively, were chosen based on literature (Frost, 1996; Prendergast et al., 1997; Turner, 1998; Claes and Heigele, 1999; Martin and Seeman, 2008). The values for m_B_^M^ and m_B_^R^, relating the bone maturation and resorption functions to the accumulated strain, were determined based on preliminary studies to achieve consistent results (Frame et al., 2017).

In summary, we presented here a multi-tissue evolution computer model in a fracture healing context and validated its predictive capability in mouse and sheep late-stage fracture healing and remodeling. This model improved the understanding of bone fracture remodeling and suggested that this healing stage can be explained by an accumulation of mechanical strains, namely that the loading history over a few hours can account for observed tissue patterning and remodeling. Previously, the model has been shown to explain bone remodeling in the femoral head (Frame et al., 2018) and in the mandible (Schmitt et al., 2016). Future work should take advantage of this model to investigate impaired healing situations or non-unions, e.g., due to osteoporosis or other co-morbidities, and evaluate the validity of the model in this context. Moreover, different fixation or implant-based systems could be tested to evaluate their healing potential in specific situations.

## Data Availability

The raw data supporting the conclusion of this article will be made available by the authors, without undue reservation.

## Abbreviations

⊗, Tensor product; B (subscript), Bone; C (subscript), Cartilage; Δ, Laplacian; D, Diffusion tensor; E, Young’s modulus; $\varepsilon_{k}$ , Principal strain (*k* = I, II, III); $\varepsilon_{k,N}$ , Normalized principal strain (*k* = I, II, III); exp, Exponential function; F (subscript), Fibrous tissue; $f_{i,k}$ , Tissue response to principal strain *k* (*i* = B, C, F); I (superscript), Immature; I, Identity tensor; $\phi_{i}$ , Tissue fraction [tissue type *i* = B, C, F or tot (total)]; M (superscript), Mature; ν, Poisson’s ratio; $\theta_{k}$ , Principal stress *k* direction; $t_{act,i}$ , Strain accumulation for tissue *i*; $t_{act,i}^{bound}$ , Bounded strain accumulation response for tissue *i*; $T_{i}$ , Tissue-specific response after restriction to activation range; $T_{i}^{G}$ , Tissue formation function for tissue *i*; $T_{i}^{M}$ , Maturation function for tissue *i*; $T_{i}^{R}$ , Resorption function for tissue i.
