# Supplementary material for: A 3D in Silico Multi-Tissue Evolution Model Highlights the Relevance of Local Strain Accumulation in Bone Fracture Remodeling
Source: Front Bioeng Biotechnol. 2022 Mar 31;10:835094. doi: 10.3389/fbioe.2022.835094 (PMC9008279; doi:10.3389/fbioe.2022.835094)
Supplement: Supplementary file 1 [file DataSheet1.pdf]

Supplementary Material

1 Model parameter sensitivity analysis

1.1 Methods

56 different parameters were used in the multi-tissue evolution model. To identify their relative contribution to the simulation outcome, a parameter sensitivity analysis was performed with three levels for each parameter: -1 (divided by 2), 0 (baseline value) and +1 (multiplied by 2). This analysis was performed on a simplified geometry: a 3D beam with a square section of side 30mm and length 90mm subjected to a loading of 1 MPa applied at the free end of the beam, the other one being clamped (Supplementary Figure 1). Initial tissue fractions were defined homogeneously in the beam as follows: 40% mature bone and 10% of each other immature and mature tissue fractions. Tetrahedral elements of average size 3.5 mm were used. 3 different ROIs were defined: lower, middle and upper 10-mm horizontal slice in the beam (Supplementary Figure 1).

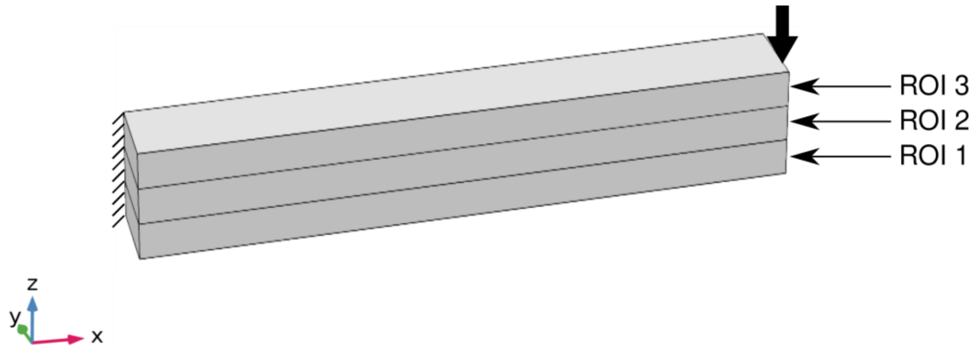

Supplementary Figure 1. Geometry and boundary conditions used for the parameter sensitivity analysis with region of interest (ROI) definition.

Based on a Taguchi array, 243 simulation were run, from which the individual contribution of each parameter could be derived by calculating the sum of squared deviations (SSD) on the output value of interest: the average Young's modulus in the upper, middle and lower third ROI after 2500 and 5000h simulation time. Taking the Young's modulus into account allowed to integrate the various tissue types into one unique output value.

1.2 Results: Most influential parameters after 5000h

The 7 most influential parameters for the Young's modulus average values in the different ROIs after 5000h were: the bone yield strain, $b_{B,III}$ and $c_{B,III}$ (coefficients relating the bone mechano-response to the third principal strain), $b_{B,I}$ and $c_{B,I}$ (coefficients relating the bone mechano-response to the first principal strain), m_B^M (a bone maturation function coefficient), m_B^R (a bone resorption function coefficient). The bone yield strain was derived from an experimental study (Bayraktar et al., 2004) and was therefore assumed to be known.

To further investigate the effect of the other 6 parameters, 2 simulations were run for 10000h: a "low-level" simulation (1) where all 6 parameters had their value leading to reduced Young's moduli and a "high-level" simulation (3) where all 6 parameters had their value leading to increased Young's moduli

(Supplementary Table 1). Those 2 simulations were compared to the baseline case (2) (all parameters taken as defined in the baseline computer model). Supplementary Figure 2a-c shows the Young's modulus distribution in the mid-section of the beam after 10000h: as expected, the "low-level" simulation led to a reduced Young's modulus (Supplementary Figure 2a) compared to the baseline case (Supplementary Figure 2b). On the contrary, the "high-level" simulation predicted much more bone formation leading to high Young's modulus values in all 3 ROIs (Supplementary Figure 2c). In Supplementary Figure 3, the average Young's modulus is depicted over time, revealing the long-term very different values.

Supplementary Table 1. Parameter values for the baseline simulation (2) and the additional simulations (1), (3), (4) and (5)

**Parameter \ simulation**	**1**	**2**	**3**	**4**	**5**
m_B^M (s)	5000	10000	20000	5000	20000
m_B^R (s)	20000	10000	5000	10000	10000
$b_{B,I}$ (s^{-1})	3.125	6.25	12.5	6.25	6.25
$b_{B,III}$ (s^{-1})	3.75	7.5	15	7.5	7.5
$c_{B,I}$ (s^{-1})	-1.125	-0.5625	-0.28125	-0.5625	-0.5625
$c_{B,III}$ (s^{-1})	-1.5	-0.75	-0.375	-0.75	-0.75
l_B^M (s)	30000	30000	30000	60000	15000
k_B^M (s)	1	1	1	0.5	2
k_B^R (s)	1	1	1	2	0.5
α_B ($m^3.s^{-1}$)	4E-6	4E-6	4E-6	2E-6	8E-6
γ_B ($m^3.s^{-1}$)	2E-6	2E-6	2E-6	1E-6	4E-6

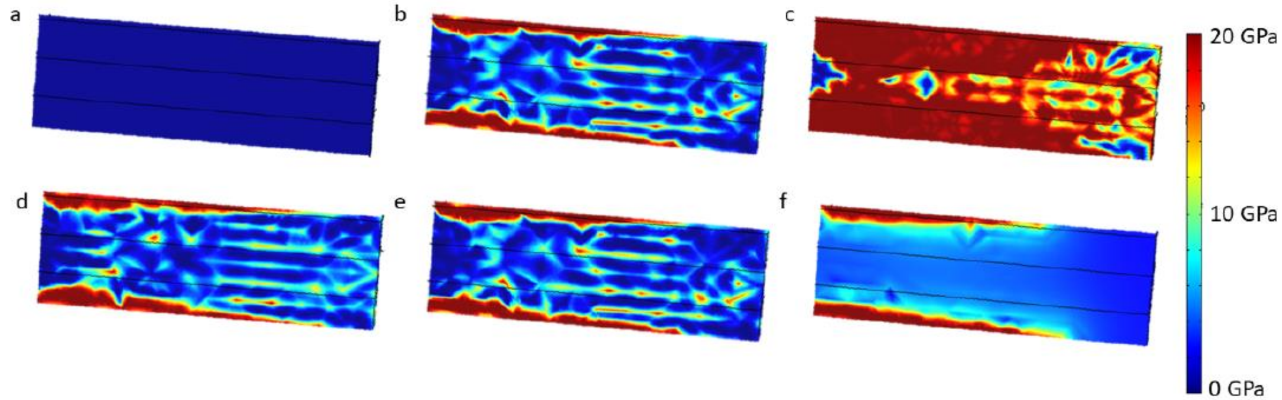

Supplementary Figure 2. Young's modulus distribution in the mid-section of the 3D beam after 10000h simulation time for: (a) simulation (1), (b) simulation (2), (c) simulation (3), (d) simulation (4), (e) simulation (2) and (f) simulation (5). The Young's modulus colour scale is given on the right.

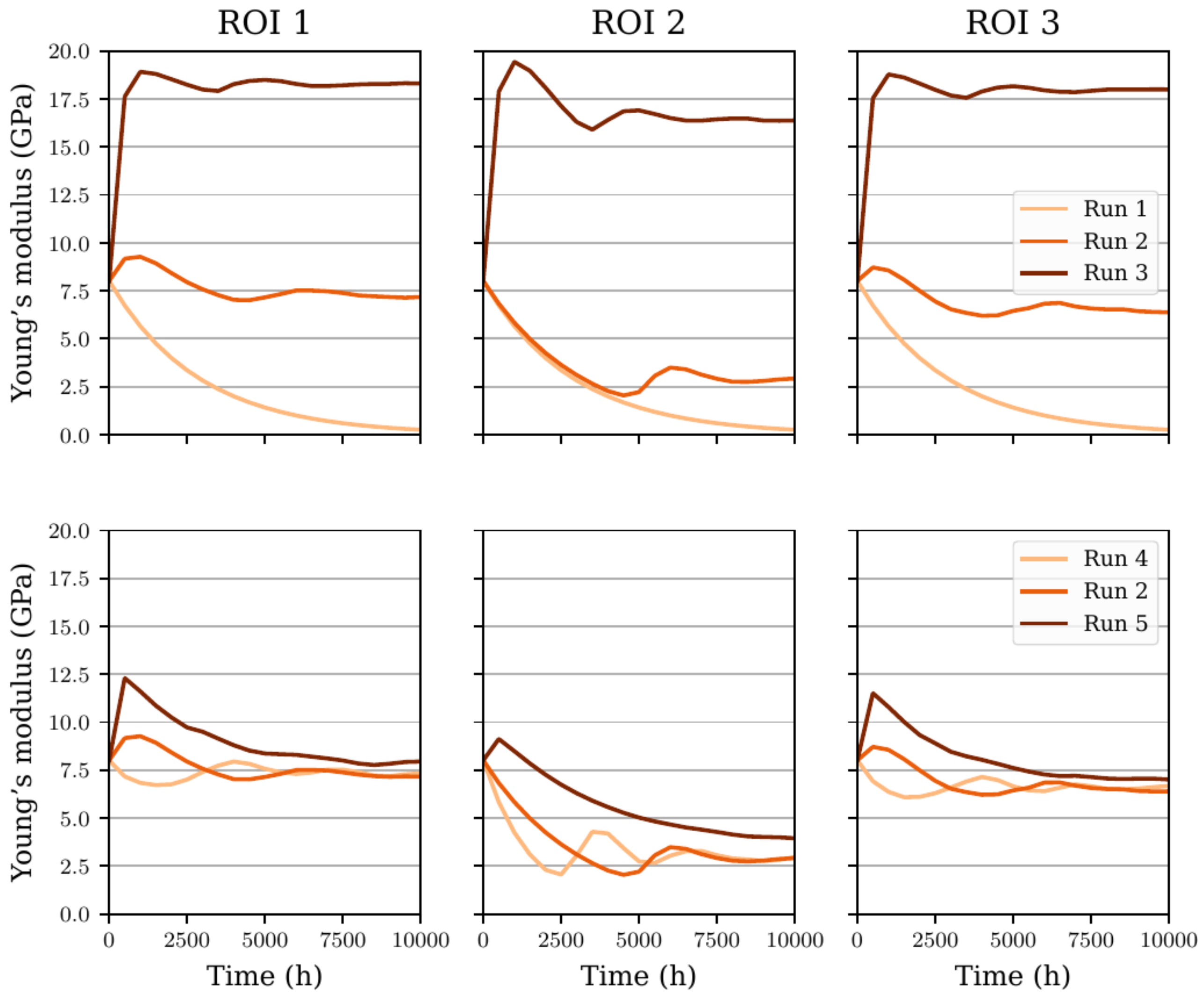

Supplementary Figure 3. Average Young's modulus time evolution in each ROI for simulations (1) to (3) (first row) and (2,4,5) (second row).

1.3 Results: Most influential parameters after 2500h

The 6 most influential parameters for the Young's modulus average values in the different ROIs after 2500h were: m_B^M, l_B^M, k_B^M (the 3 coefficients of the bone maturation function), k_B^R (a bone resorption function coefficient), the bone formation rate α_B and the bone maturation rate γ_B.

To further investigate the effect of those 6 parameters, 2 simulations were run for 10000h: a "low-level" simulation (4) where all 6 parameters had their value leading to reduced Young's moduli and a "high-level" simulation (5) where all 6 parameters had their value leading to increased Young's moduli (Supplementary Table 1). Those 2 simulations were compared to the baseline case (2) (all parameters taken as defined in the baseline computer model). In those cases, longer-term predictions did not differ much between the baseline case and the high- or low-level simulations (Supplementary Figure 2d-f); only the dynamics of the prediction was different (Supplementary Figure 3).

1.4 Conclusions

The parameter sensitivity analysis revealed that the simulations are mostly sensitive to a few bone mechano-response and maturation or resorption coefficients at the longer-term (equilibrium state): $b_{B,III}$ and $c_{B,III}$, $b_{B,I}$ and $c_{B,I}$, m_B^M, m_B^R . The values for $b_{B,III}$, $c_{B,III}$, $b_{B,I}$ and $c_{B,I}$ were chosen based on literature (Frost, 1996; Prendergast et al., 1997; Turner, 1998; Claes and Heigele, 1999; Martin and Seeman, 2008). The values for m_B^M and m_B^R were determined based on preliminary examples to achieve consistent results (Frame et al., 2017). The same values were therefore employed for the study described here.

2 References

Bayraktar, H. H., Morgan, E. F., Niebur, G. L., Morris, G. E., Wong, E. K., and Keaveny, T. M. (2004). Comparison of the elastic and yield properties of human femoral trabecular and cortical bone tissue. *Journal of Biomechanics* 37, 27–35. doi:10.1016/S0021-9290(03)00257-4.

Claes, L. E., and Heigele, C. A. (1999). Magnitudes of local stress and strain along bony surfaces predict the course and type of fracture healing. *Journal of Biomechanics* 32, 255–266. doi:10.1016/S0021-9290(98)00153-5.

Frame, J., Rohan, P.-Y., Corté, L., and Allena, R. (2017). A mechano-biological model of multi-tissue evolution in bone. *Continuum Mech. Thermodyn.* doi:10.1007/s00161-017-0611-9.

Frost, H. M. (1996). Perspectives: a proposed general model of the "mechanostat" (suggestions from a new skeletal-biologic paradigm). *Anat Rec* 244, 139–147. doi:10.1002/(SICI)1097-0185(199602)244:2<139::AID-AR1>3.0.CO;2-X.

Martin, T. J., and Seeman, E. (2008). Bone remodelling: its local regulation and the emergence of bone fragility. *Best Pract Res Clin Endocrinol Metab* 22, 701–722. doi:10.1016/j.beem.2008.07.006.

Prendergast, P. J., Huiskes, R., and Søballe, K. (1997). Biophysical stimuli on cells during tissue differentiation at implant interfaces. *Journal of Biomechanics* 30, 539–548. doi:10.1016/S0021-9290(96)00140-6.

Turner, C. H. (1998). Three rules for bone adaptation to mechanical stimuli. *Bone* 23, 399–407. doi:10.1016/S8756-3282(98)00118-5.
